# Effect of Black Grape Juice against Heart Damage from Acute Gamma TBI in Rats

**DOI:** 10.3390/molecules181012154

**Published:** 2013-09-30

**Authors:** Robson Borba de Freitas, Aline Augusti Boligon, Bruno Tomazele Rovani, Mariana Piana, Thiele Faccim de Brum, Roberta da Silva Jesus, Fagner Chagas Rother, Nelson Mendes Alves, João Batista Teixeira da Rocha, Margareth Linde Athayde, Juan Pablo Barrio, Edson Ramos de Andrade, Liliane de Freitas Bauerman

**Affiliations:** 1 Postgraduate Program in Pharmaceutical Sciences, Center of Health Sciences, Federal University of Santa Maria (UFSM), Santa Maria, RS, 97105-900, Brazil; 2 Nuclear Engineering Program, Military Institute of Engineering (IME), Rio de Janeiro, RJ, 22290-270, Brazil; 3 Postgraduate Program, Institute of Nuclear and Energy Research of University of São Paulo (IPEN), São Paulo, SP, 05508-000, Brazil; 4 Department of Chemistry, Center of Natural and Exact Sciences, Federal University of Santa Maria (UFSM), Santa Maria, RS, 97105-900, Brazil; 5 Department of Industrial Pharmacy, Center of Health Sciences, Federal University of Santa Maria (UFSM), Santa Maria, RS, 97105-900, Brazil; 6 Department of Biomedical Sciences and Institute of Biomedicine (IBIOMED), University of León, León 24071, Spain; 7 Brazilian Army Technological Center (CTEx), Chemical, Biological and Nuclear Defense Division, Rio de Janeiro, RJ, 23020-470, Brazil; 8 Department of Physiology and Pharmacology, Center of Health Sciences, Federal University of Santa Maria (UFSM), Santa Maria, RS, 97105-900, Brazil

**Keywords:** acute radiation syndrome, black grape juice, heart, oxidative damage, lactate dehydrogenase, phenolics, flavonoids, HPLC-DAD

## Abstract

The aim of this study was to evaluate the potential positive effect of black grape juice (BGJ) on lipid peroxidation considering Total Body Irradiation (TBI) in Wistar rats. As a potential feasible means of evaluation *in situ*, blood serum lactate dehydrogenase (LDH) levels were evaluated as a marker for heart damage from acute radiation syndrome (ARS). Twenty rats were divided into four groups, two of them being irradiated by gamma-rays from a Co-60 source. Animals were treated by gavage with 2 mL per day of BGJ or placebo for one week before and 4 days after 6 Gy whole body gamma-irradiation, when they were euthanasiated. LDH on serum and lipid peroxidation on heart tissue were evaluated. High concentration of metabolites from lipid peroxidation in heart, and high LDH level on serum were found only in gamma-irradiated group given placebo, mainly at the first 24 h after radiation. Phytochemical analysis of BGJ was performed by determining total phenolics, flavonoids, and tannins followed by a high-performance liquid chromatography (HPLC/DAD) analysis, which showed resveratrol as the major constituent. Results suggest that BGJ is a good protective candidate compound against heart damage from ARS and its effects suggest its use as a radiomodifier.

## 1. Introduction

Ionizing radiation can be defined as an electromagnetic radiation or a high-energy particle capable of depositing its energy or a fraction when it interacts with atoms leading to ionization [1]. Pathophysiological consequences of the interaction of ionizing radiation with biological systems may come either from direct and indirect modes. The direct mode radiation will directly hit an atom or molecule in the cell probably leading to a collision and producing poisoning fragments. On the other hand, the indirect mode supports the model of radiation energy transfer directly to water molecules producing oxidizing metabolites such as singlet oxygen (^1^O_2_), superoxide anion (O_2_^●^) and hydroxyl radical (•OH). In this case, the result is a possible cell death or malfunction due to the interactions between biomolecules and reactive oxygen species (ROS) and nitrogen reactive species (NRS) [2]. Free radicals and, ROS are capable of damaging the cell membrane and proteins at the nucleus. This situation may change the functional activity of the cell with a possible further death pathway or undesirable organic states [3].

Acute radiation syndrome (ARS) is a representative set of symptoms and signals that is deterministically related to the acute whole body exposure to ionizing radiation. Radiation damage results from cell sensitivity to radiation exposure [4]. Differences in cell sensitivity to radiation are the basis for the distinction among the three subsyndromes in ARS: hematopoietic, gastrointestinal, and neurovascular [4]. As radiation doses increase subsyndromes become detectable separately from each other. Each subsyndrome can be further divided into four stages: (a) prodromal, (b) latent, (c) manifest illness, and (d) recovery or death. Since prodromal symptoms could be detected from few h to 4 days after exposure, the earlier the evaluation of the absorbed dose, the better the response to medical care will be [5].

The heart is a vital organ and generates intense oxidative imbalances because of its intense activity. Moreover, the heart presents a less potent antioxidant system when compared to other body tissues. As an example, the catalase (CAT) activity in heart tissue is due prioritarily to erythrocyte catalase [6]. Studies have shown that heart tissue exposure to ionizing radiation may induce coronary and valvular heart disease, congestive heart failure and sudden death [7]. This situation can be linked to the broad range of oxygen free radicals produced from interactions with ionizing radiation, since the ROS are the major mediators for radiation-induced damage in living tissues.

During a heart attack caused by stress from ARS, the cardiomyocyte plasmatic membrane lose its fluidity, causing extravasations of cytosolic enzymes (transaminases, alkaline phosphatase and lactate dehydrogenase) to circulating blood, indicating a pathological condition. Serum biochemical analysis, like lactate dehydrogenase (LDH) activity could provide a trustful way to estimate radiation damage to specific organs such as heart [8,9]. LDH converts pyruvate, the final product of glycolysis to lactate when oxygen is absent or in short supply as happens in heart tissue physiology. Because of this, measuring stress from oxygen-poor environments under radiation allow one to infer some conclusions about the major aspects of damage in well oxygenated tissues. 

Total body X-irradiation in rats led to a marked increase in total LDH activity in serum and tissues [10]. Thyagarajan and colleagues observed that the maximum effect was recorded at the 8th post-irradiation day. LDH levels in serum are mentioned in the literature as a marker of heart tissue harm from ARS and, such enzyme measurement may be an alternative way to quantify the risk of harm due to ARS in general, and, at heart specifically without intrusive testing [11]. 

Compounds capable of preventing or mitigating damage as a result of exposure to radiation are called radiomodifiers and have been studied [12,13,14,15,16]. These substances possess the property of reducing the effect of ionizing radiation considered due to damage from free radicals and ROS formed once radiation interacts with water, especially by catalyzing the ROS and, NRS generated in the radiation-tissue interaction. Clinically relevant radiomodifiers usually have low or no toxicity and synergy with other drugs. A good alternative to be available quickly for use as radiomodifier would be the use of food supplements rich in bioactive compounds with antioxidant action. Foods that contain such compounds are called functional foods or nutraceuticals [17].

Flavonoids and phenolic compounds scavenge oxidizing species produced by radiation and elevate the enzymatic and non-enzymatic defense levels [18]. The radioprotector effect of these compounds appears to be improved when acting synergistically. This evidence was proved by Lee and colleagues who investigated the radioprotector effect of *Camellia sinensis* (green tea) aqueous extract and its isolated polyphenols (epicatechin, epigallocatechin, epigallocatechin gallate, gallocatechin gallate, cathequin gallate) in mice [19]. The results showed a significant increase in endogenous spleen colonies in irradiated mice pre-treated with green tea in comparison to those pre-treated with the isolated compounds [19].

An overall nutrigenomic action of a nutraceutical or a functional food occurs through the interaction of food compounds with the genome as a whole or through its interaction with specific genes [20]. Grape products, especially wine, have been investigated for their functional or nutraceutical properties regarded to radiation damage alleviation with important results, such as mitigation of anorexia [15].

Studies based on epidemiological and experimental evidences showed that the polyphenolic compounds present in grapes-derived products, including juices, and in particular quercetin and resveratrol [21,22], have antioxidant properties [23], antitumor activity [24,25], antiinflammatory [26], antimutagenic [27], hepatoprotective [28], cardioprotective [29], and radioprotective [12] among other interesting activities.

To date, studies regarding compounds and dietary products from black grape and its positive radiomodifying effect are limited to the effect of polyphenols extracted from grape seed extract (GSE) [13]. Results from grape seed extract reported by Saada and colleagues suggest that radioprotective effects or lessened radiotoxicity are possible [13]. These authors found significant increases of superoxide dismutase (SOD), catalase (CAT) and glutathione peroxidase (GSH-Px) activities associated with significant decreases of malondialdehyde (MDA) levels, a product formed during the lipid peroxidation process, in the heart tissue of rats pre-treated with GSE and exposed to 5 Gy of gamma-rays in comparison to irradiated control group [13]. The hypothesis that a moderate intake of black grape juice (BGJ) also has a positive radiomodifier property against heart damage following ARS is an important issue to be investigated.

## 2. Results

### 2.1. Phenolics, Flavonoids and, Condensed Tannins Contents and, HPLC-DAD Analysis in Black Grape Juice (BGJ)

**Figure 1:**
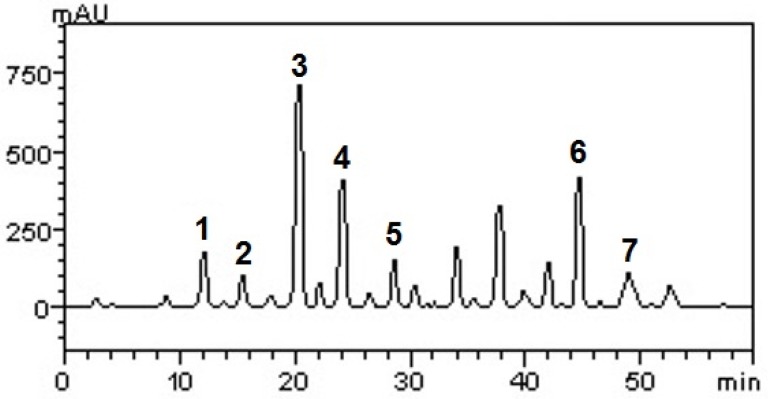
Representative high performance liquid chromatography (HPLC-DAD) profile of BGJ, detection UV was set at 327 nm. Gallic acid (peak 1), catechin (peak 2), resveratrol (peak 3), caffeic acid (peak 4), ellagic acid (peak 5), quercetin (peak 6) and kaempferol (peak 7).

Data on related phenolics, flavonoids and, condensed tannins contents are given in Table 1*.* HPLC fingerprinting of BGJ revealed the presence of gallic acid (t_R_ = 11.87 min; peak 1), catechin (t_R_ = 15.69 min; peak 2), resveratrol (t_R_ = 20.03 min; peak 3), caffeic acid (t_R_ = 24.11; peak 4), ellagic acid (t_R_ = 28.37 min; peak 5), quercetin (t_R_ = 44.81 min; peak 6) and kaempferol (t_R_ = 49.16 min; peak 7) (Figure 1 and Table 1). The concentration for each substance is given in Table 1. Also, we show the corresponding chromatogram in Figure 1.

**Table 1:** Content of phenolics, flavonoids and condensed tannins of BGJ. LOD and LOQ variations for antioxidants compounds.

BGJ compounds	Composition mg/L	LOD mg/mL	LOQ mg/mL
Total phenolics (GE)	223.14 ± 0.005	-	-
Total flavonoids (Quer)	180.45 ± 0.011	-	-
Total tannin (Cat)	154.09 ± 0.017	-	-
Gallic acid	8.43 ± 0.06^a^	0.017	0.056
Catechin	5.17 ± 0.09^b^	0.032	0.104
Resveratrol	31.28 ± 0.04^c^	0.009	0.030
Caffeic acid	18.15 ± 0.03^d^	0.013	0.041
Ellagic acid	8.03 ± 0.05^a^	0.028	0.093
Quercetin	18.67 ± 0.12^d^	0.015	0.049
Kaempferol	8.71 ± 0.03^a^	0.008	0.026

GE: gallic acid equivalent, Quer: quercetin equivalent, Cat: catechin equivalent. LOD: limit of detection (mg/mL), LOQ: limit of quantification (mg/mL). Results are expressed as mean ± standard deviations (SD) of three determinations. Averages followed by different letters differ by Tukey test at *p* < 0.05.

### 2.2. LDH Levels

**Figure 2:**
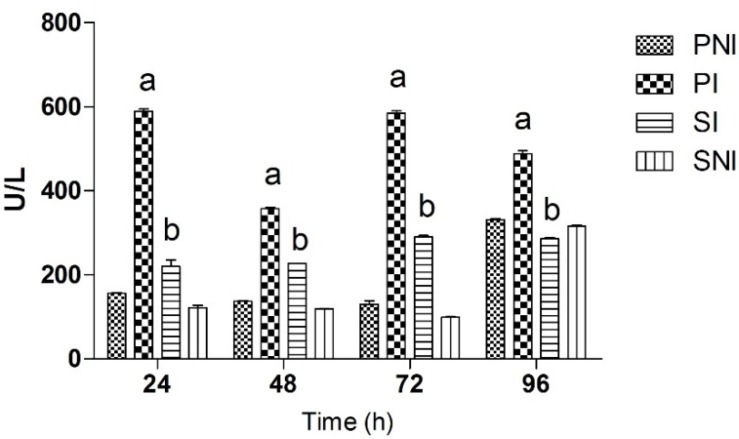
Serum lactate dehydrogenase (LDH) levels for all groups of male Wistar rats at 24, 48, 72 and 96 h after exposure to radiation.

As can be seen in Figure 2, the LDH levels were statistically meaningful for comparison between SI (irradiated animals and supplemented with BGJ) and PI (irradiated animals and supplemented with placebo solution). Control groups PNI (non-irradiated animals and supplemented with placebo) and SNI (non-irradiated animals and supplemented with BGJ) values remained normal and statistically indistinguishable from each other until 72 h, with a slight increase at 96 h. Overall, PI showed higher levels of LDH in comparison with SI.

The legends represent the following groups: (SNI) non-irradiated, BGJ supplemented; (PNI) non-irradiated, placebo (isocaloric glucose plus fructose) supplemented; (SI) irradiated, BGJ supplemented, and (PI) irradiated, placebo supplemented. **a** and **b** means significant differences for PI and SI. Values are expressed as means ± S.E.M. for n = 5. Labels **a** and **b** mean significant difference between groups for *p* < 0.05.

### 2.3. Lipid Peroxidation on Heart

**Figure 3:**
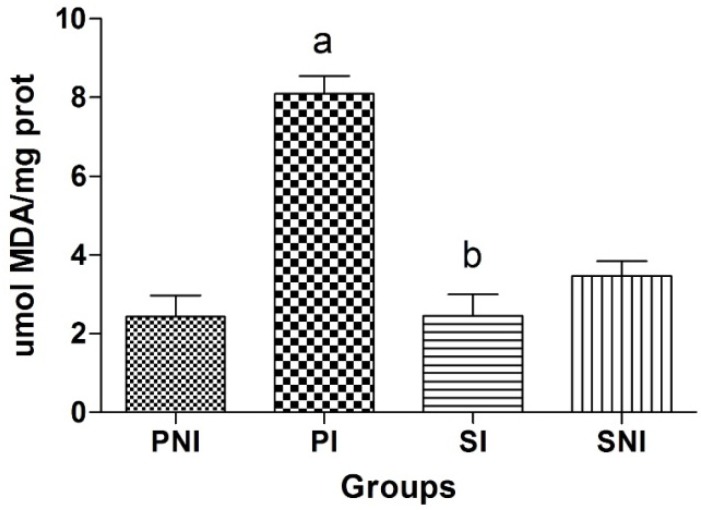
Thiobarbituric acid reactive substances (TBARS) levels expressed in µmol MDA/mg protein.

Lipid peroxidation was estimated by measuring the MDA per mg of heart muscle protein for each animal in each group, using the thiobarbituric acid reactive substances (TBARS) assay. The greater the damage, the greater the MDA formation. The highest MDA index of heart was found in PI group as expected. Figure 3 shows the estimation of lipid peroxidation in heart tissue for all groups. A significant difference between PI and SI compared each other was found with higher MDA levels for PI. SI was kept at basal levels in comparison to non-irradiated groups (PNI and SNI), possibly due to BGJ intake and its positive radiomodifier effect against oxidative damage.

The legends represent the following groups: (SNI) non-irradiated, BGJ supplemented; (PNI) non-irradiated, placebo (isocaloric glucose plus fructose) supplemented; (SI) irradiated, BGJ supplemented, and (PI) irradiated, placebo supplemented. There is no significance for SI in comparison to PNI and SNI. Values are expressed as means ± S.E.M for n = 5. Labels **a** and **b** mean significant difference between groups for *p* < 0.05.

## 3. Discussion

Ionizing radiation exposure caused by environmental, industrial, medical and accidental reasons can make the organism susceptible to various harmful conditions [30]. The whole body exposure a high dose of radiation also called the acute radiation syndrome (ARS) or radiation sickness, and might cause alterations in the hematopoietic [16], cardiovascular [31] and digestive systems besides disturbance in biochemical parameters [32]. Various studies were designed to prospect positive radiomodifiers which might reduce the incidence of injuries related to ARS, among them, medicinal plants, vitamins and, minerals. Natural compounds in the human diet could provide functional antioxidants, like vitamin E [33] and grape derived products, such as wine and juices [12]. In grapes, four classes of ﬂavonoids are commonly detected: ﬂavonols, anthocyanins, ﬂavan-3-ols and their polymeric forms, and condensed tannins [34]. Concerning phytochemical analysis, colorimetric assays confirmed the presence of phenolics, flavonoids and condensed tannins in BGJ as previously described by Machado and colleagues [35]. Interestingly, in 2013, we found high concentration of condensed tannins in BGJ, approximately 770 times higher than the quantity found in BGJ in 2007. This fact can be justified by the variations in phytochemical components of grapes which are common when collected in different periods. The major component of BGJ, detected by HPLC-DAD, was found to be resveratrol, followed by quercetin, rutin, caffeic and gallic acids, kaempferol, ellagic acid and, catechin. Resveratrol (3,5,40-trihydroxy-*trans*-stilbene) is a polyphenolic phytoallexin found in a variety of plant products [36]; it is a potent antioxidant, and has garnered interest because its protective effects in complex conditions such as ARS [16]. Additionally, the literature describes the correlation between phenolic compounds such as caffeic acid and low incidence of heart damage.

LDH dosage in blood serum is currently used to evaluate the risk of diseases such as heart attack and could be used as predictive factor for heart tissue harm due to radiation over exposure [37].

LDH levels were found significantly increased on animal’s serum in PI and SI when compared with controls (Figure 2). This behavior agrees with previous studies, which reported that radiation caused significant increases in creatine phosphokinase (CPK) and LDH [31]. Hence, the excessive production of free radicals and lipid peroxides might cause the leakage of cytosolic enzymes including the aminotransferases, creatine kinase and phosphatases enzymes [31].

However, the information that SI group showed significant reduced levels of LDH on 24 h after radiation when compared to PI is suggestive of an immediate action of BGJ intake. This profile remained unchanged on 48, 72 and, 96 h after ARS induction. Various studies relate the low incidence of heart diseases and the intake of food and beverage containing phenolic compounds and, flavonoids [38]. Caffeic acid presents a great radiomodifier effect in rats exposed to 7 Gy gamma whole-body radiation [31]. The putative mechanism of action of this substance and other phenolics resides in its antioxidant capacity, reducing the oxidative damage on heart tissue induced by water radiolysis. Phenolic and flavonoids found in BGJ are great antioxidants because their molecular structures contain hydroxyl groups which provide electrons to stabilize free radicals and ROS, restoring the redox state of cardiomyocytes.

High MDA levels can be indicative of oxidative damage to mitochondria and heart muscle cell membranes. It is possible that oxidative damage caused by irradiation could promote cell death processes due to membrane damage such as radiation-induced apoptosis [39,40].

Significant differences in lipid peroxidation for PI and SI (Figure 3) allow us to infer that the phenolics contents of BGJ, principally resveratrol, and its action as scavenger of hydroxyl radicals, might be the reason for the protection offered to the membrane and reduction of MDA formation in the SI group. Overall, changes in MDA levels can be a signature of changes in membrane physiology, and BGJ seems to protect against this physiological imbalance [16]. Compounds in BGJ, exemplified by quercetin and resveratrol, have been investigated extensively and there is evidence that they can activate transcription factors that regulate the expression of genes encoding for antioxidant enzymes [41]. These enzymes remove peroxides and hydroperoxides formed during radiation intoxication, attenuating oxidative processes in membrane lipids and the proteins of heart cells. 

The protection against heart lipid peroxidation offered by BGJ intake might have an effect towards amelioration of serum LDH levels in ARS condition. Although these results are in agreement with recent reports [12,15] which support the idea that phenolics and flavonoids present in BGJ effectively act against ARS effects in rats, they are on an *ad hoc* basis and need further investigation before extrapolating to humans.

## 4. Experimental

### 4.1. Black Grape Juice (BGJ) and Placebo Solution

Ecologically-produced (organic) BGJ was obtained from the city of Garibaldi (Rio Grande do Sul, Brazil), in the main grape-growing region of the state. Grapes (*Vitis labrusca*) were cultivated in 2013 and the juice was prepared on the same year. Placebo solution was made using an equimolar mixture of glucose and fructose to be isocaloric with the sugar composition in the BGJ (95 g/L).

### 4.2. Colorimetric Assays

#### 4.2.1. Determination of Total Phenolics

The determination of total phenolic content was performed by the Folin-Ciocalteu method [42]. Briefly, 2 N Folin-Ciocalteu reagent (0.5 mL) was added to BGJ (1 mL) and this mixture was allowed to stand for 5 min before the addition of 20% Na_2_CO_3_ (2 mL). The solution was then allowed to stand for 10 min before reading at 730 nm in a Shimadzu-UV-1201 (Shimadzu, Kyoto, Japan) spectrophotometer. The phenolics content was expressed in milligrams of gallic acid equivalents per liter of BGJ. The equation obtained for the standard curve of gallic acid was y = 52.167x − 0.0631 (r = 0.9999). The experiments were performed in triplicate.

#### 4.2.2. Determination of Total Flavonoids

The determination of the flavonoids content was performed according to the method described by Woisky and Salatino [43]. To BGJ (2 mL) was added a solution of AlCl_3_ (2%, 0.5 mL). After 15 minutes, the absorbance was read at 420 nm. Tests were performed in triplicate and for calculating the dosage of flavonoids used the standard curve of quercetin was: y = 0.0457x − 0.009 (r = 0.9997). Concentrations of flavonoids were determined in milligrams equivalents of quercetin per liter of BGJ. The experiments were performed in triplicate.

#### 4.2.3. Determination of Condensed Tannins

The tannin content was performed using the method described by Morrison *et al.* with some modifications [44]. BGJ (1 mL), solution A (1 g of vanillin in 100 mL of methanol) and solution B (8 mL of HCl in 100 mL of methanol) were used for assay. Sample was read at 500 nm on a spectrophotometer. The tannin total was expressed as milligram equivalents of catechin per liter of BGJ. The equation obtained for the calibration curve of catechin was y = 0.0309x − 0.065 (r = 0.9989). The experiments were performed in triplicate.

### 4.3. High Performance Liquid Chromatography

#### 4.3.1. Chemicals, Apparatus and General Procedures

All chemicals were of analytical grade. Methanol, acetic acid, gallic acid, caffeic acid and ellagic acid purchased from Merck (Darmstadt, Germany). Catechin, resveratrol, quercetin and kaempferol were acquired from Sigma Chemical Co. (St. Louis, MO, USA). High performance liquid chromatography (HPLC-DAD) was performed with a Shimadzu Prominence Auto Sampler (SIL-20A) HPLC system (Shimadzu, Kyoto, Japan), equipped with Shimadzu LC-20AT reciprocating pumps connected to a DGU 20A5 degasser with a CBM 20A integrator, SPD-M20A diode array detector and, LC solution 1.22 SP1 software.

#### 4.3.2. Quantification of Compounds by HPLC-DAD

Reverse phase chromatographic analyses were carried out under gradient conditions using C_18_ column (4.6 mm × 150 mm) packed with 5 μm diameter particles; the mobile phase was water containing 2% acetic acid (A) and methanol (B), and the composition gradient was: 5% (B) for 2 min; 25% (B) until 10 min; 40, 50, 60, 70 and 80% (B) every 10 min; following the method described by Kamdem *et al.* with slight modifications [45]. The BJG was filtered through 0.45 μm membrane filter (Millipore) and then degassed by ultrasonic bath prior to use. The flow rate was 0.6 mL/min, injection volume 50 μL and the wavelength was 254 for gallic acid and resveratrol, 280 nm for catechin, 327 nm for caffeic and ellagic acids, and 365 nm for quercetin and kaempferol. The samples and mobile phase were filtered through 0.45 μm membrane filter (Millipore) and then degassed by ultrasonic bath prior to use. Stock solutions of standards references were prepared in the HPLC mobile phase at a concentration range of 0.010–0.100 mg/mL for resveratrol, catechin, quercetin and kaempferol; and 0.050–0.200 mg/mL for gallic, ellagic and caffeic acids. The chromatography peaks were confirmed by comparing its retention time with those of reference standards and by DAD spectra (200 to 600 nm). Calibration curves were for gallic acid: y = 14036x + 1254.9 (r = 0.9991); catechin: y = 12,724x + 1,258.0 (r = 0.9997), resveratrol: y = 11,983x + 1,306.5 (r = 0.9993); caffeic acid: y = 11,872x + 1,570.3 (r = 0.9996); ellagic acid: y = 12,728x + 1,367.4 (r = 0.9998); quercetin: y = 13,149x + 1267.8 (r = 0.9999) and kaempferol: y = 15,983x + 1,321.5 (r = 0.9992). All chromatography operations were carried out at ambient temperature and in triplicate.

#### 4.3.3. Limit of Detection (LOD) and Limit of Quantification (LOQ)

LOD and LOQ were calculated based on the standard deviation of the responses and the slope using three independent analytical curves of phenolics compounds and flavonoids, as defined by Boligon *et al*. [46]. LOD and LOQ were established as 3σ/S and 10 σ/S, respectively, where σ is the standard deviation of the response and S is the slope of the calibration curve.

### 4.4. Animals and Whole Body Irradiation

Twenty male Wistar rats weighing 200–250 g, housed at the animal house of Nuclear Energy Research Institute (IPEN-Brazil), were included in the study. Animals were divided into four groups: non-irradiated-BGJ supplemented (SNI, n = 5); non-irradiated–placebo supplemented (PNI, n = 5); irradiated-BGJ supplemented (SI, n = 5) and irradiated–placebo supplemented (PI, n = 5). In order to immobilize the animals, anesthesia was induced by intraperitoneally administration of pentobarbital 0.6% in saline (10 mL/kg body weight), at noon, 15 minutes before irradiation, ensuring the loss of palpebral and plantar reflex activity and checking for spontaneous respiration throughout the procedure. The animals were placed in *decubitus pronus* on a plexiglas board, so that five animals would be irradiated at a time and exposed to a single dose of 6 Gy (0.40 Gy/min) of whole body gamma-irradiation, with a source-skin distance (SSD) of 50 cm. Animals were fed with BGJ or placebo (2 mL/Kg of body weigth, by gavage) for one week before and 4 days after 6 Gy whole body gamma-irradiation. The experimental protocol used was approved by the Nuclear Energy Research Institute under the protocol number 048/09–CEPA–IPEN/SP.

### 4.5. Food and Drink

Animals were fed according to a standard rat chow diet, having free access to *ad libitum* water and food. After one week adaptation to individual cages, they were daily fed with 2 mL of BGJ or placebo, depending on their assigned group. Environmental conditions were controlled (12-hour photoperiod and 20 ± 2 °C) throughout the experimental period.

### 4.6. Blood Samples Collection and LDH Measurement

The blood samples for LDH measurement from twenty male Wistar rats were obtained at 24, 48, 72 and 96 h following exposure to gamma radiation using heparinized capillaries by puncturing the retro-orbital plexus after prior mild anesthesia with isofluoran. After the last blood sample collection, all animals were euthanized and heart was removed for analyses. LDH was assayed in a Cobas MIRA^®^ automatic analyzer (Roche Diagnostics, Basel, Switzerland) with the Bioclin^®^ commercial systems.

### 4.7. Lipid Peroxidation of Heart Tissue

Heart tissue lipid peroxidation estimation was performed using the thiobarbituric acid reactive substances assay (TBARS) as described by Buege [47], which quantifies the colorimetric reaction of the lipid peroxidation product malondialdehyde (MDA) with thiobarbituric acid (TBA). The reaction produces a colored compound which absorbs maximally at 532 nm. One gram of heart tissue in 5 mL of potassium phosphate (0.1 M, pH 7.4) was homogenated using a Polytron mixer (Kinematica AG, Luzern, Switzerland). After heating at 90 °C for reacting with TBA, tubes were cooled and centrifuged at 2,000 × *g*. The organic layer (supernatant) was collected and the absorbance was read at 532 nm using a spectrophotometer.

### 4.8. Quantification of Heart Protein

The amounts of lipid peroxidation were normalized to the amount of heart protein contained by measuring the MDA per mg of heart protein. The quantification of the protein was performed following the Lowry method, where the maximum absorbance for the Folin Ciocauteau solution due to its interaction to bovine serum albumin protein, occurs at 625 nm [48].

### 4.9. Statistical Analysis

For lipid peroxidation in heart tissue and LDH, statistical significance was assessed by using the Student-Newman-Keuls Multiple Comparisons Test by GraphPad® Prism 5 software (GraphPad Software, San Diego, CA, USA, August 2007). For HPLC-DAD fingerprint of BGJ, statistical comparisons were performed by one-way analysis of variance followed Tukey’s *post-hoc* test. Values are expressed as means ± S.E.M. A value of *p* < 0.05 was considered statistically significant.

## 5. Conclusions

Blood biochemical measurement (LDH) and lipoperoxidation index regarding heart tissue (MDA formation) support the idea that BGJ has a positive radiomodifier effect against heart dysfunction due to ARS. The evidence for BGJ this effect against heart tissue damage can be listed as follows: (a) lipid peroxidation on SI group kept at basal levels; (b) dramatic reduction of LDH levels in blood serum for SI groups. This way, we conclude that BGJ as a functional beverage is a good candidate for investigation as a positive radiomodifier agent against heart complications due to ARS because it is rich in resveratrol, quercetin, caffeic acid and others. Further investigation on damage to heart tissue due to oxidative stress caused by exposure to radiation should be performed, including the primary antioxidant system.
